# Principles in practice? A policy review of the IOC's environmental sustainability agenda

**DOI:** 10.3389/fspor.2025.1511092

**Published:** 2025-02-11

**Authors:** Alison Cain, Michael Callan

**Affiliations:** Institute of Sport, Department of Psychology, Sport and Geography, University of Hertfordshire, Hatfield, United Kingdom

**Keywords:** environmental sustainability, policy, strategy, critical policy discourse analysis, International Olympic Committee, power, greenwashing, unintended consequences

## Abstract

This paper is a policy review focused on the environmental sustainability (ES) agenda of the International Olympic Committee (IOC). This incorporates exploration of IOC documents such as policies, strategies, guidelines, reports, codes, and conference outputs. The IOC's ES agenda is interpreted as both strategy around ES, as a plan of action to achieve desired outcomes, and policies around ES, as matters of practice and principle to be enacted. This review encompasses each of the IOC's three spheres of activity (as an organisation, as owner of the Olympic Games, and as leader of the Olympic Movement). The documentary analysis incorporates inductive thematic analysis and Critical Policy Discourse Analysis (CPDA). This allows for consideration of the role of the IOC as a driver of ES with the power and reach to influence pro-environmental behaviours on a global scale, as well as analysis of the extent to which documentary discourse demonstrates committed leadership in this sphere. Key themes arising from the data are networks and knowledge transfer, leadership and influence, governance and accountability, and opportunities and obstacles. Power relationships between stakeholders are important in terms of facilitating or inhibiting ES, and there are missed opportunities for the IOC both to better demonstrate positive ES outcomes from existing practices and to utilise its power in leveraging ES commitments from stakeholders across the Olympic Movement (OM). The application of CPDA highlights varying levels of commitment across these themes as well as a tendency toward ambiguity and contradiction that engenders the likelihood of unintended consequences including greenwashing. The IOC ES agenda needs to encompass clear and unambiguous policy and strategy with more explicit commitment and accountability across its three spheres of activity.

## 1 Introduction

It is widely accepted that we are living in a climate emergency, with record air temperatures being recorded (1), increasingly unpredictable weather patterns and events (2), and the hottest ever ocean temperature (3). Increasingly, climate change is dominating the news and there is growing recognition from individuals, organisations, and governments of the need for behaviour change involving environmental sustainability (ES). Sports organisations are no exception to this. Indeed, the President of World Athletics, Lord Sebastian Coe, has stated that “climate change should increasingly be viewed as an existential threat to sport” (4, p. 4). Concerns over climate change and a growing focus on ES are increasingly being recognised within multiple and varied sports contexts, including the Olympic Games (OG) (5). The social significance of sport suggests the OG can be a meaningful platform for promoting ES on a global scale, while hosts of the Games can lead the ES agenda in global sport, highlighting ways in which sport can have a positive impact on the environment and encouraging pro-environmental behaviours through an educative approach. This highlights the importance of the IOC leveraging its influence in seeking impactful ES objectives from OG hosts and ensuring these are fulfilled (6), which requires coherent and committed policy that is clear and unambiguous such that it can be enacted effectively (7). Yet, talk has been shown to fill an action vacuum with policy discourse remaining as words rather than being translated into credible and meaningful enactment. The IOC has for many years sought to establish itself as a credible actor in the realm of ES (8), as discussed in the context section below, yet scepticism remains over its legitimacy. Thus, an examination of policy in terms of both content and discourse can aid understanding of the authenticity of commitment to policy enactment.

The core purpose of this paper is to provide a review of the IOC's ES agenda, as detailed in a range of policy and strategy documents produced by the organisation that address its three spheres of activity (as an organisation, as owner of the Olympic Games, and as leader of the Olympic Movement). The latter two spheres in particular highlight the powerful position occupied by the IOC in world sport. Thus, this policy review also identifies how the IOC's documented ES agenda may harness the organisation's leadership role to leverage ES outcomes in the Olympic Movement (OM). The OM comprises a range of Olympic stakeholders and so consideration is given to how the IOC's documented ES agenda may be subject to varying interpretations by these stakeholders. Through this review, policy implications and actionable recommendations are identified.

In this paper, the definition of ES is based on the Brundtland Report's definition of sustainable development as that which “meets the needs of the present without compromising the ability of future generations to meet their own needs” (9), p. 7). This definition has been chosen from the multiple definitions available as it is clear, concise, widely used in literature on sport and the environment, and remains the most commonly referenced definition of sustainable development (10). It is applied here with a specific focus on the environment.

## 2 Context

The significance of sport in the fight against climate change was made clear at COP26 (26th Conference of Parties, in Glasgow in 2021) with greater involvement of sports organisations and athletes than at any previous COP (11). Over 280 sport federations signed up to the United Nations' Race to Zero campaign, which is aimed at reducing carbon emissions (12). The IOC has pledged commitment to this campaign and the broader UN Sports for Climate Action Framework, which are at the forefront of the IOC's ES initiatives (13). Thus, the IOC claims that it “is walking the talk on sustainability” (14) and so can exhibit leadership in ES, particularly as the role of sport in enabling the UN's Sustainable Development Goals (SDGs) has been identified as significant (15).

As a whole, the sports industry is a contributor to climate change through fan and athlete travel, stadium and infrastructure construction, energy use, and more (16). At the same time, sports accept sponsorship from organisations that are arguably even more significant contributors to climate change and, in so doing, help to legitimise those companies and conceal the ecological harm they cause. This is greenwashing (16) and an example of this is the IOC acceptance of Toyota as one of its TOP (The Olympic Partner) programme sponsors. Toyota supported sustainability with mobility solutions at the Tokyo Games and supports with reducing the emissions impact of the IOC vehicle fleet (17). Despite this apparent commitment to sustainable mobility, it is difficult to reconcile this with an organisation that produces approximately 10 million vehicles annually that are sold in 170 countries. Toyota's partnership with the IOC therefore serves to help legitimise the company's activities and offers a means of promoting its sustainability claims. That said, the Olympic Agenda 2020 Closing Report refers to a hydrogen powered fleet of Toyota cars that are used by the IOC, which indicates that there are environmental sustainability benefits to the partnership (18). However, it has been reported that Toyota is ending its contract with the IOC (19). Similarly, the Dow Chemical Company was a TOP sponsor that was identified as the official carbon partner of the IOC and that has been frequently cited in IOC documentation as playing an important part in the IOC's sustainability strategy (20). The link between organisations with environmentally damaging core business (e.g., Toyota's vehicles, Dow's chemicals, Bridgestone's tyres) and the positive values of Olympism has been apparent ever since the Olympic Movement's Agenda 21 framework, embraced by the IOC and the OM in 1999, was published with support from Shell (21). Another TOP sponsor, Coca-Cola, has faced growing criticism for not reducing its plastic and has been accused of subterfuge for dispensing drinks from plastic bottles in reusable cups at the Paris Games (22). Single-use plastics, transportation and food waste are three of the main problems still associated with large-scale sports events, and so it is difficult to reconcile genuine commitment to ES intentions without evidence of meaningful action to address these issues. The IOC, arguably the most influential institution across a range of sports globally, has moved from a focus on the environment to a broader focus on sustainability in recent years, exemplified by replacing the Sport and Environment Commission with the Sustainability and Legacy Commission. Sustainability as a concept is widely accepted as encompassing three dimensions, namely environmental, social and economic, which have been referred to as the *Triple Bottom Line* (TBL) (23). Whilst the IOC's shift in emphasis toward sustainability may enable the organisation to capture social and economic matters within its strategic objectives, the focus of this paper is on the environmental dimension since this is the aspect most clearly linked to climate change.

According to Cantelon and Letters (24), the environment was incorporated as the third pillar of Olympism in 1996 (the first two pillars being sport and culture). This has been well documented elsewhere and it is not the purpose of this paper to revisit it further here. There is a wealth of literature pertaining to the IOC and environmental sustainability, much of it sceptical about the sincerity of the IOC's declared commitment to this realm. Questions over the IOC's sincerity in relation to ES commitments have been raised over many years, indeed ever since the organisation began delving into this sphere. Since the literature on this is so extensive, two examples of such questioning are presented here as illustrative of the views that IOC green claims are more performative than authentic.

Mincyte et al. (25) contended that environmentalism has been inextricably linked with the Olympic brand since Sydney 2000. Despite Sydney 2000 being dubbed the “Green Games” and branded as an environmentally and ecologically focused event, it was criticised for greenwashing (16, 26, 27). Beder (28) claimed that government promises of a “green” Games in Sydney 2000 were a spurious marketing ploy rather than being authentic.

Lesjø (29, p. 292) traced such showcasing by the IOC further back, to the planning for Lillehammer 1994, describing the IOC's engagement with the environmental agenda as “instrumental”, i.e., a means to an end, and the environmental Olympics slogan as a “marketing strategy” that was not embraced by the IOC in any meaningful way.

The Olympic brand is not only attached to hosts of the Games but is formulated and championed by the IOC as the governing body of the OM. Evidence indicates that greenwashing may apply to the IOC's stated commitment to ES that is not necessarily corroborated by action (30–33). This is supported by McCullough et al. (34) who suggested that there is an emphasis placed by the IOC on ES during the bidding phase but this tends not to be enforced once a host has been selected. However, recent changes to the process for awarding the hosting rights could address this issue (17, 35). In addition, in recent years, a growing range of evidence has emerged that suggests the IOC is, as it claims, *walking the talk*. For example, ES attributes were built into the design and function of Olympic House (the IOC's headquarters in Lausanne, Switzerland). In addition, the IOC's involvement in recent years in the Sport Positive Summit (the biggest annual event focused on ES for global stakeholders in sport) may be evidence that the organisation is internalising its green claims and growing in legitimacy (36).

Yet, the fact remains that unless there are mandatory targets or benchmarks, rather than just recommendations (31), and a system of sanctions for not meeting these, there will remain a lack of accountability not only for failing to meet ES-related goals but also for any negative environmental impacts arising from hosting the Games. Since IOC sustainability strategy is underpinned by the idea of sport as an enabler of pro-environmental behaviour change and is informed by the IOC relationship with the UN in relation to sustainability and climate, it is important to assess the viability of the IOC's strategy and broader ES agenda (encompassing policies, practices and plans) in terms of the potential for genuine impact on ES and climate change mitigation.

## 3 Method

This review incorporated qualitative document analysis (37, 38), comprising elements of inductive content analysis, policy analysis, discourse analysis and reflexive thematic analysis (37, 39).

Critical policy analysis is used widely in research into sustainability as well as research on manifestations and the operation of power. Similarly, Critical Discourse Analysis (CDA) has been identified as fitting for the examination of organisations involved in global governance within a given field, particularly in terms of power relations (40), and useful for analysing unintended consequences and environmental discourse (41). For example, Boykoff and Mascarenhas (31) utilised CDA in examining the bid books for Rio 2016. A policy review approach incorporating CDA thus addresses the need for critique of discourses of environmental sustainability surrounding the OG, as identified by McLeod et al. (42).

Mulderrig et al. (43) advocate integration of CDA with Critical Policy Studies (CPS) to produce an approach defined as Critical Policy Discourse Analysis (CPDA). CPDA synergises theory and methodology into an analytical framework that contributes to policy research through a conceptualisation of textual details in relation to the ways in which these details can affect the implementation of policy (43). The utility of this approach for the purpose of the present study is clear, in that the aims are to analyse the IOC agenda (policy and strategy) on ES through textual analysis that also seeks to consider how the discourse used may affect the enactment of policy across the IOC's three spheres of responsibility. Policy is dynamic and is not only what is documented but what is enacted, and a focus on enactment facilitates better understanding of context (44).

### 3.1 Sampling

A long list of documents (*n* = 84) comprising policies, strategies, codes of practice, reports, frameworks, manuals, fact sheets and conference outputs was initially identified through purposive sampling using web-based searches, starting with the IOC website, and subsequently following a snowball approach as links led to additional sources. Documents referred to in relevant literature were also included. The long list included any documentary material that was accessible, written in English and related to ES and the IOC. In addition, documents that were identified on the Olympic Studies Centre (OSC) website but were unavailable online were requested for supervised consultation at the OSC Library and reviewed there during a visit to Lausanne.

Following exclusion of duplicates, criteria were developed for the purpose of shortlisting the remaining documents to select an appropriate analysis sample, paying attention to the four factors for sampling identified by Flick (45); authenticity, credibility, representativeness and meaning (see Table 1).

**Table 1:** Inclusion and exclusion criteria.

Criteria	Inclusion	Exclusion	Flick's factors for sampling
Date range	2012–2021	Pre-2012, post-2021	Representativeness
Access	Full text availability	No/partial availability	Meaning, authenticity
Language	English	Non-English	Credibility
Source	IOC	Non-IOC	Credibility, authenticity
Relevance (specific reference to keywords)	≥30 references to keywords, contextually verified	<30 references to keywords, contextually unrelated	Meaning (importance of documents as signifiers of ES policy, strategy, intent, aims, objectives, action, outcomes)

**Table 2:** The final shortlist of IOC documents in the order they were analysed.

Document name	Total keywords	Date of publication	Justification for inclusion
10th World Conference on Sport and Environment Final Report (46)	349	2013 (Oct-Nov)	The first of these conferences to take place within the date range for inclusion (and the last to date)
IOC Annual Report 2014 (47)	77	2014	First annual report since publication of Olympic Agenda 2020 and first in this format (previous report covered 2009–2012)
IOC Sustainability Strategy (48)	411	2017 (October)	Key document (includes IOC Sustainability Policy)
Olympic Games the New Norm Report (35)	31	2018 (February)	Covers the six recommendations that focus on the OG (from the 40 identified as part of Olympic Agenda 2020)
Host City Contract (HCC) Operational Requirements (49)	114	2018 (June)	This document is contractually binding for OG hosts and was the first step in implementation of the New Norm
IOC Sustainability Report 2018 (17)	759	2018 (October)	The first IOC sustainability report following publication of the sustainability strategy
IOC Sustainability Report 2021 (50)	299	2021	The final sustainability report to be issued during the date range for inclusion, which includes a closing report for 2017–2021
IOC Annual Report 2021 (13)	212	2021	Final annual report in included date range

The selected date range for the shortlisted document sample (2012–2021 inclusive) covers the period since the development of Olympic Agenda 2020 (and its adoption in 2014) and the publication of the most recent IOC Sustainability Report (2021). Proceedings of the 10th World Conference on Sport and the Environment were deemed to be an appropriate starting point as this can be seen as fundamental in the development of Agenda 2020 and was also held at the start of Thomas Bach's tenure as President of the IOC, who has overseen Agenda 2020.

NVivo was used for shortlisting with all long-listed documents imported into a project. Two text search queries were applied using the query wizard. Although some query features are not available in the wizard tool, this was unproblematic given the straightforward requirements of this initial stage of shortlisting. The search terms used were “environment” and 'sustainable’ and the NVivo query slider tool was set to include stemmed words such that the search would also yield results for related terms such as “environmental” and “environmentalism” and “sustainability.” The slider was not set to find similar concepts since both search terms have more than one meaning depending on the context in which they are used, thus a search for similar terms might have yielded irrelevant results (for example, surroundings, situation, maintainable, workable).

The next step of the shortlisting process was a manual check, which had two purposes. First, since NVivo search cannot find words in some formats, manual checking using the “Find text” tool in Adobe Acrobat allowed for confidence that no documents were erroneously excluded for low frequency of keywords. Second, the manual check ensured the frequency results were not skewed by contextually irrelevant references to keywords, which further addressed “meaning” as a factor in the sampling process (37, 45).

Morgan (37) explained how sample selection for qualitative document analysis is an iterative process that may require repeat exploration of possible documents until such time as the sample for inclusion reaches saturation, i.e., inclusion of additional documents would not provide any further data themes. The manual processes undertaken facilitated repeat exploration and further ensured the representativeness of the sample. The final shortlist (see Table 2) was judged to satisfy the sampling criteria.

### 3.2 Analysis

NVivo was not used for the coding and analysis, with a manual process preferred to enable reflexive thematic analysis combined with CPDA that allows for contextualisation rather than a narrower, uncontextualised approach.

Analysis itself was an iterative process involving multiple reviews of the documents. The initial phases of inductive thematic content analysis involved open coding, generation of first order concepts and identification of over-arching themes. Open codes were extracted and grouped by concept and by theme. These themes were then analysed using a CPDA approach, which focused on the discourse used when ES objectives, initiatives and outcomes were discussed. Use of specific verbs, inferences, provisos and so forth serve to contextualise the policy statements, for example use of “will” conveys greater commitment than use of “should”. This approach allows for discernment of the perceived level of commitment underpinning ES intentions or expectations with the documents.

## 4 Results and discussion

Four overarching themes emerged from the thematic analysis: Networks and Knowledge Transfer, Governance and Accountability, Influence and Leadership, and Opportunities and Obstacles.

There are several first-order concepts associated with each of these themes, which are presented in Table 3.

**Table 3:** Themes and first-order concepts.

Over-arching theme	Associated first-order concepts
Networks and Knowledge Transfer	Partnership and collaboration Stakeholder involvement Synergies and coordination Expertise and experience Innovation and solutions Consultation and feedback Sharing and applying best practice
Governance and Accountability	Regulations, standards, codes, and compliance Policies, practices, processes, and plans Certifications, licences, and independent assurance Targets, measurement, monitoring, and mitigation Implementation: Analysis, evaluation, and review Transparency and reporting Resource allocation for sustainability matters
Influence and Leadership	Vision, values, and principles Reach, recognition, and leverage Catalysts and exemplars Credibility Communication, promotion and showcasing Education, training, advocacy, and awareness-raising Shaping attitudes and changing behaviours
Opportunities and Obstacles	Drivers and incentives Aims, aspirations and intentions Expectations and obligations Tangible and intangible outcomes and impacts Caveats and conflicts of interest Challenges Limitations

There is a great deal of repetition within and across documents, which helps both justify the representativeness and saturation of the sample and suggests there may be a lack of substance with which to populate the documents such that reiteration of rhetoric is used to flesh out the content. A salient example of such fleshing out can be found on page 25 of the Sustainability Strategy, which comprises various facts and figures from the Rio 2016 Games with no apparent relevance to sustainability nor strategy and no contextualisation. Such content was excluded from analysis.

### 4.1 Results by theme and concepts

In Tables 4–7 below, one pertinent excerpt from the document sample is presented to exemplify each first-order concept. The level of repetition meant that most of the illustrative excerpts appeared in multiple documents, and those presented are representative of the concepts and themes.

**Table 4:** Illustrative excerpts for the theme of networks and knowledge transfer.

First-order concept	Illustrative excerpt
Partnership and collaboration	“The IOC has also developed – and will continue to develop – strategic partnerships with major global organisations in the field of sustainability [e.g., United Nations (UN) Environment, International Union for the Conservation of Nature]. Their own networks will enable the IOC to extend its reach to promote the role of sport and Olympic values in sustainable development”
Stakeholder involvement	“The five focus areas reflect aspects of the IOC's activities that have the most significant interaction with sustainability. They have also been selected by considering today's key sustainability challenges and the manner in which the IOC and its stakeholders believe the IOC can most effectively contribute”
Synergies and coordination	“During the development of the IOC Sustainability Strategy…we consulted with many international organisations to identify potential synergies, areas of interest and concrete actions that would assist us in responding to Olympic Agenda 2020 and implementing the Strategy. Identifying and consulting with potential partners is an ongoing process – in many cases, these partner organisations are able to provide assistance to OCOGs [Organising Committee of the Olympic Games] and host cities, either directly or via their international networks”
Expertise and experience	“TOP Partners…will benefit the IOC's sustainability work. Their experience, products and technologies can be leveraged to increase the impact of the Sustainability Strategy”
Innovation and solutions	“Facilitate exchanges between Olympic Games stakeholders (e.g., OCOGs, national partners, host city authorities, TOP partners) and build strategic partnerships with relevant expert organisations to develop innovative sustainable solutions”
Consultation and feedback	“Stakeholder consultation to develop the IOC Sustainability Strategy. Over 100 stakeholders and experts consulted: IOC Members and IOC staff, IFs, NOCs, OCOGs, 2024 candidate cities, TOP partners…Over 25 international organisations [Including the United Nations Environment Programme (UNEP), the International Union for the Conservation of Nature (IUCN), the International Labour Organisation (ILO), WWF, the World Union of Olympic Cities, Green Sports Alliance, etc] and sustainability experts”
Sharing and applying best practice	“Olympic Games London 2012 raised the bar to new levels and it has been the IOC's goal to have subsequent Olympic Games build on these foundations … The effect has gone beyond the Olympic Games to influence the wider event sector. An example is the Event Sustainability Management System standard, ISO 20121, which was pioneered by the Olympic Games London 2012. [ISO 20121] has rapidly become the recognised international sustainability standard for events and is now a requirement for Organising Committees for the Olympic Games (OCOGs)”

**Table 5:** Illustrative excerpts for the theme of governance and accountability.

First-order concept	Illustrative excerpt
Regulations, standards, codes, and compliance	“Belonging to the Olympic Movement requires compliance with the Olympic Charter. The Olympic Charter is the codification of the Fundamental Principles, Rules and Bye-laws adopted by the International Olympic Committee. It governs the organisation and running of the Olympic Movement and sets the conditions for the celebration of the Olympic Games”
Policies, practices, processes, and plans	“IOC Sustainability Strategy will fundamentally shape the working practices of the IOC, the Olympic Games and the Olympic Movement. IOC defines the product scope, scale, programme and operational requirements … and sets the contractual rules of engagement (Host City Contract)”
Certifications, licences, and independent assurance	“Initial scope of the IOC Sustainability Management System will cover the planning and operation of institutional events (e.g., IOC Session meetings, IOC Commission meetings, Olympism in Action Forum). (This excludes the Olympic Games since ISO 20121 certification is the responsibility of the OCOG, as required under the terms of the Host City Contract)”
Targets, measurement, monitoring, and mitigation	“The implementation of the Sustainability Management System will rely, in particular, on … processes and tools, including Key Performance Indicators (KPIs) to measure progress towards our objectives”
Implementation: Analysis, evaluation, and review	“Strengthen support and monitoring of the OCOGs’ implementation of sustainability-related bid commitments, Host City Contract requirements and IOC's recommendations, including through the provision of common methodologies and independent third party assessments where appropriate”
Transparency and reporting	“The timing of this first Sustainability Report is in accordance with the commitment in our Sustainability Strategy to report every two years, approximately six months after the completion of the preceding Winter or Summer Olympic Games…Meanwhile, we will provide progress updates and related information on a regular basis via the sustainability pages on olympic.org. This will include shorter, summary annual Sustainability Reports, highlighting material changes and results related to the implementation of our Strategy”
Resource allocation for sustainability matters	“The Director General … oversees the … allocation of resources – including for the delivery of the Sustainability Strategy …The implementation of the Sustainability Management System will rely, in particular, on … financial, human and technical resources that will be defined in annual action plans”

**Table 6:** Illustrative excerpts for the theme of influence and leadership.

First-order concept	Illustrative excerpt
Vision, values, and principles	“Following the development of Olympic Agenda 2020, the Olympic Movement's vision statement…was updated and approved by the IOC Session in 2015. It included for the first time explicit reference to sustainability as a working principle of the Olympic Movement. This recognised the need to move from a technical approach to one where sustainability is integral to the culture of the organisation”
Reach, recognition and leverage	“We aim to use our global reach to bring the sustainability work of Olympic Movement stakeholders to a broader audience … we intend to leverage two major assets: Olympic Solidarity, the established main mechanism through which the IOC supports the National Olympic Committees (NOCs), and Olympic athletes, who can provide the inspiration and profile to excite greater attention in this area”
Catalysts and exemplars	“Through a game-changing collaboration, Dow and the IOC are using the platform of sport and the Olympic brand to catalyse action on climate change and help build a blueprint for a more sustainable future”
Credibility	“Although the impact of our own corporate activities is small compared to the Olympic Games or the Olympic Movement, it is vitally important that we as an organisation “walk the talk” to be credible towards our partners”
Communication, promotion, and showcasing	“The publication of all the [sustainability] guides has been widely communicated via IOC digital channels, the media as well as directly to the stakeholders. On average, each guide has been downloaded 1,192 times”
Education, training, advocacy, and awareness-raising	“As leader of the Olympic Movement, our strategic intent is two-fold: firstly, to engage internally within the Olympic Movement to advocate higher standards of sustainability within the sport sector; and secondly, to serve as a representative on behalf of the Olympic Movement to highlight the sustainability achievements of the sporting world to external stakeholders and to utilise the power of athletes and the Olympic brand to attract attention, create conversations and inspire interest in sustainability through sport”
Shaping attitudes and changing behaviours	“We encourage staff to use sustainable transportation modes for their daily commuting (walking, cycling, public transport). This is done by subsidising either public transportation or the purchase of a bicycle, and by providing bicycle storage spaces, changing rooms and showers at our offices. As a result, more than half of IOC staff use sustainable transportation modes for their daily commutes”

**Table 7:** Illustrative excerpts for the theme of opportunities and obstacles.

First-order concept	Illustrative excerpt
Drivers and incentives	“No two cities are alike and there can be no direct comparison between competing claims and promises. What matters is sustainability in the host territory context: an Olympic Games concept that fits with the direction in which the city/region is already heading, and that can enhance and accelerate planned programmes and help address contemporary social, economic and environmental issues”
Aims, aspirations and intentions	“Olympic Agenda 2020 places great emphasis on incorporating sustainability in all aspects of the Olympic Games and encourages all stakeholders of the Olympic Movement to include sustainability in their daily operations”
Expectations and obligations	“To define its long-term strategic sustainability approach, the IOC needs to take account of its roles and activities in each of these spheres [IOC as an organisation, IOC as owner of the Olympic Games, IOC as leader of the Olympic Movement] and the relative degrees of control and influence it can bring to bear, as well as its obligations in taking forward the sustainability recommendations from Olympic Agenda 2020”
Tangible and intangible outcomes and impacts	“To a greater or lesser degree, our policies and activities touch upon all the SDGs. However, we have particularly identified 12 SDGs to which we feel we can contribute in meaningful and tangible ways”
Caveats and conflicts of interest	“Our primary focus is to reduce carbon emissions. However, we recognise that our main source of impact, air travel, cannot be easily reduced in the current context and depends to a large extent on the locations of future Olympic Games. This means for the foreseeable future, we will still be responsible for a significant amount of travel-related carbon emissions, hence the need to consider meaningful carbon compensation options”
Challenges	“While sport is the unifying thread across the Olympic Movement, its constituents are hugely diverse in terms of size, resources, technical understanding, regulatory context, geography and culture. This makes it challenging to provide a consistent level of service. Inevitably, therefore, we must offer more tailored support to our stakeholders, which in turn has implications in terms of our human resources, budgets and timelines for delivering on our objectives”
Limitations	“We have selected the content for this report so it reflects progress towards implementing our Sustainability Strategy, as well as topics addressed by other IOC programmes and processes that are relevant to sustainability”

#### 4.1.1 Networks and knowledge transfer

This theme is about working with and learning from others to innovate and share solutions and best practice. Knowledge transfer has been a key element of the IOC approach to the sharing of good practice between former and future hosts, however the documents show that the involvement of a range of stakeholders has grown and that collaboration, innovation and the sharing of expertise in relation to ES occur between these, the IOC and hosts of the Games.

It may be appropriate and valuable to make use of the expertise and experience of TOP partners, and as claimed this could enhance the impact of the Sustainability Strategy. There are examples that the IOC draws upon, such as Dow's work with Sochi and Coca-Cola's environmental efforts, but there have also been significant criticisms of these. The IOC claim that the “goal to achieve carbon neutrality was fulfilled – and even exceeded – via projects implemented as part of the IOC-Dow global carbon mitigation programme during 2017–2021” (50, p. 14), but this has been questioned. IOC endorsement of such companies in terms of ES encourages greenwashing and has the unintended consequence of legitimising organisations that are actually contributors to environmental degradation and climate change, in which the IOC then becomes complicit. As Glasson and Hutchins (51, p. 12) conclude, partnerships such as that between the IOC and Dow serve to promote a “social imaginary of collaborative eco-capitalism”.

Similarly, evidence of stakeholder consultation appears positive, especially the involvement of organisations such as UNEP, the IUCN, World Wide Fund for Nature (WWF) and Green Sports Alliance (GSA), however what is not stated is how differing perspectives were accommodated and whose voices carried more weight when conflicts of interest arose. For example, TOP partners are likely to have very differing priorities to those of the aforementioned organisations and the balance of power may favour the partners who have made significant economic contributions (52).

The primacy of TOP partners is made explicit in the documents, with reference to it being essential that the OG are a good investment for them and that their expectations are fulfilled regarding certain aspects. This is moderated with reference to respect for the interests of OCOGs, but nonetheless it arguably indicates a balance of power that favours economic factors, which would create potential for conflicts of interest. The IOC is reportedly seeking its first TOP sponsor from India (53), a heavily polluting nation, which further raises the possibility of economic factors trumping environmental commitments.

In terms of OM stakeholder involvement in the OG, the IOC strategy now includes the transfer of certain activities to IFs, NOCs, and TOP partners, ostensibly to improve the support for OCOGs. However, it is not clear what the rationale for this transfer of responsibility is and, since it is likely to reduce the OCOG autonomy and affect power balances, it could lead to conflicts of interest and unintended consequences that inhibit ES (e.g., where a TOP partner is involved with activities where environmental targets conflict with their own objectives that may result in ES being sidelined). Furthermore, this transfer of responsibilities contradicts the statement in the sustainability strategy that “the real delivery of sustainability performance is the job of the host city and the OCOG” (48, p. 24). Any potential transfer of responsibilities to an IF is based on nine factors, the ninth of which is sustainability/environmental risks, which may imply the perceived importance of ES relative to other factors.

Overall, the Sustainability Strategy is inherent with contradictions. The claim is that control and influence diminish from one sphere of responsibility to the next (as an organisation, as owner of the OG and as leader of the OM), yet the impact grows. At the same time, achievements in the OG are implied to be IOC achievements. There is a problematic tendency in the documents where it is implied that ES achievements in the OG are IOC successes yet simultaneously for the IOC to distance itself from accountability and ownership. For example, evidence of knowledge transfer and sharing of best practice, such as the development of ISO20121, was led by the London OCOG (LOCOG) but has been claimed by the IOC as evidence of its own sustainability achievements. It is good that the IOC now require ISO20121 to be adhered to by OCOGs, but it is not an IOC achievement. It will be interesting to see if a similar pattern emerges with the supplier code, since the IOC developed its code following the introduction of a sourcing code by the Tokyo OCOG (TOCOG).

In terms of sharing best practice, there is evidence in the documents of the IOC downplaying involvement in overseeing ES initiatives post-Games. This is disingenuous as it disregards the former Olympic Games Global Impact (OGGI) programme and fails to refer to the more recently developed Legacy Reporting Framework (LRF). Although the claim is made that greater involvement with hosts post-Games is envisaged, this is not framed in committed terms and is intended to be leveraged through collaboration with the World Union of Olympic Cities (UMVO) rather than being led by the IOC itself.

Generally, there is a tendency throughout the documents for caveats to be presented that moderate the IOC's responsibility in terms of ES deliverables. A clear example of this is in the following extract from the Sustainability Strategy:

As owner of the Olympic Games, the IOC defines the product scope, scale, programme and operational requirements … and sets the contractual rules of engagement (Host City Contract). Following the host city election, the IOC's role is essentially one of contract management to oversee and support the local Organising Committee of the Olympic Games (OCOG), while the real delivery of sustainability performance is the job of the host city and the OCOG (48, p. 14).

The IOC defines the operational requirements and sets the contractual rules, so it is perplexing for it to not also share responsibility for delivery, e.g., through mechanisms (and sanctions) to ensure compliance.

#### 4.1.2 Governance and accountability

This theme focuses on embracing regulatory frameworks, taking responsibility, and ensuring compliance. The extracts presented in Table 5 are indicative of the IOC approach to governance of ES in its three spheres of responsibility (as an organisation, as owner of the OG, and as leader of the OM). Taking a CPDA approach to analysis, the language used in this theme shows less commitment than is evident across the other themes.

The fact that compliance with the Olympic Charter is a requirement for being part of the OM offers a clear opportunity for the IOC to mandate ES requirements as part of this. The Olympic Charter states that “[t]he IOC's role is…to encourage and support a responsible concern for environmental issues, to promote sustainable development in sport and to require that the Olympic Games are held accordingly…” (54, p. 14). The extract from the Olympic Charter is of particular importance as it is included in the IOC's Sustainability Strategy yet apparently is not invoked in any meaningful way to ensure ES is embedded throughout the OM. Furthermore, the strategy section entitled *Making It Happen*, which addresses the governance of sustainability, should be a key section but lacks specificity. The content is all quite vague, with reference made to a dedicated sustainability team and a core implementation team but no explanation of how these interact, who is in each and what their roles and responsibilities are.

It is suggested that successful implementation of the Sustainability Management System will be reliant on tools such as KPIs, yet nowhere in the document sample are these KPIs identified. This again lends itself to vagueness and obfuscation. In addition, implementation will be reliant on “a Sustainability Policy formalising top management commitment to sustainability principles and continuous improvement” (48, p. 46), yet the policy itself is vague and lacking genuine commitment and accountability.

There is an encouraging intention to greater robustness of monitoring OCOGs' delivery of commitments and HCC requirements, with reference to the possibility of independent evaluation. However, there is a useful caveat in the phrase “where appropriate” since this embeds ambiguity and subjectivity that is the antithesis of the objectivity associated with independent assessment. It is not made clear how such appropriateness would be determined or by whom.

There is some encouraging evidence of expectations of compliance within the wider OM, such as a new clause having been included in standard contracts for IOC licensees that refers to environmental and social obligations. Similarly, the requirement for OG from 2030 onwards to be climate positive has been added to the contract for hosts. While these examples are promising, the real test will be in how they are monitored and how the relevant stakeholders are made accountable, e.g., whether sanctions will be applied in the event of non-compliance. In both cases, there is also room for obfuscation either through an emphasis on social responsibilities over environmental commitments or through a lack of clarity over measurement of climate impacts.

In terms of reporting, further problems appear that cast doubt on the authenticity of IOC commitments. For example, in the Sustainability Strategy (2017) and the first Sustainability Report in 2018 there is a stated commitment to production of biennial reports as well as shorter annual summaries, yet there has been no report since 2021. There is a progress update from November 2019 and a report from December 2021 but no new objectives or reports/updates/strategy since then. If the stated commitment to regularity of reporting has not been adhered to, what might this imply for other commitments (that are arguably more challenging to implement)?

The 2018 Sustainability Report was produced in accordance with the Global Reporting Initiative (GRI) Standards – Core Option and was verified by an independent certification provider (ERM Certification and Verification Services). This was highlighted as a means of good practice, yet the 2021 report did not adhere to this robust approach and instead followed the format of the progress report, which is surprising given that the 2021 version was also the closing report for the quadrennial strategy and as such would have benefitted from rigour and independent assurance.

Whilst it is promising that the Director General is identified as responsible for resource allocation to implement the Sustainability Strategy, there is no information provided on how this allocation is decided. With the three spheres of activity of the IOC, as well as the TBL of sustainability, this again allows for ambiguity and obfuscation. As with the KPIs that have been referred to but not identified in relation to the Sustainability Management System, the reference to resources necessary for its implementation is not meaningful without explicit definition. These resources are apparently defined in annual action plans, yet these plans have not been issued and this highlights a lack of either transparency or authenticity.

In the Sustainability Strategy section on *Sourcing and Resource Management*, the scope only refers to the OM, which is the sphere of responsibility in which the IOC claims its direct control is limited. This is interesting since it allows the organisation to distance itself from the issues and recommendations made in this focus area. It is unclear why the other two spheres of responsibility are not included here.

#### 4.1.3 Influence and leadership

This theme reflects the importance of sport to many people in societies across the world, and the potential this importance offers for reaching a wide audience. The theme is thus categorised as harnessing the significance of sport to advocate and educate. It demonstrates the vision and values espoused by the IOC, and the capacity for sport to be used as vehicle to leverage behaviour change (e.g., the need for environmentally friendly behaviours).

The problematic distancing of the IOC from the environmental impacts of the OG and OM is crystallised in the statement that “the impact of our own corporate activities is small compared to the Olympic Games or the Olympic Movement” (48, p. 39). This is disingenuous since the IOC “owns” the OG and OM so, by extension, it owns the impact of these and could leverage its power (or ownership) to mandate the fulfilment of ES objectives. The statement continues by saying “it is vitally important that we as an organisation ‘walk the talk’ to be credible to our partners” (48, p. 39), which is somewhat contradictory since greater credibility would develop from taking appropriate responsibility as owner of the OG and leader of the OM. Credibility was one of the three pillars of Olympic Agenda 2020 (along with sustainability and youth), which together comprised 40 recommendations that the IOC claim as evidence of *walking the talk*. However, the lack of IOC accountability indicated by the policy discourse makes this appear to be empty rhetoric. Similarly, in relation to advocacy there are references to growing numbers of athletes from a range of sports speaking out and raising awareness of various issues including ES. Whilst there is plenty of evidence to support this, these efforts are not being led or coordinated by the IOC and so it is disingenuous to present such activities as though they are related to the IOC strategy or policy.

Nonetheless, the 2021 Annual Report seeks to convey a vision of “Innovation Through Collaboration” that leverages IOC influence with TOP Programme stakeholders “epitomised by greater cooperation between the IOC and the TOP Partners in key focus areas such as technology, digital and sustainability” (13, p. 131). The success of this vision, which was to guide the TOP Programme from 2021 to 2024, is yet to be demonstrated and would be influenced by the extent to which the IOC could utilise its power as leader of the OM to seek innovative sustainable solutions that are not hindered by the primacy of economic interests. Somewhat paradoxically, the report states that the impact of this strategic vision was evident at the Tokyo Games, even though these took place at the start of the time frame for its application. This offers further evidence of the aforementioned tendency identified for the IOC to lay claim to OG successes.

Furthermore, for the IOC as an organisation (the sphere in which the IOC has greatest autonomy), there are significant ES actions that could be taken in terms of corporate activities, such as holding IOC Sessions either in a fixed venue or, better still, virtually. This is discussed further in the Opportunities and Obstacles section below.

#### 4.1.4 Opportunities and obstacles

This theme encompasses the motivations that underpin a focus on ES, the intended outcomes, and the contextual factors that affect achievement of these, including barriers, challenges, conflicts of interest, and unintended consequences. Again, the application of CPDA highlights prevalence of provisos and caveats that are indicative of a lack of commitment.

Similar to its control over the siting of IOC Sessions, the IOC as owner of the OG could limit or influence the locations of future Games, thus to claim that the impact of air travel by IOC representatives is dependent on where the Games are held again highlights the artificial distancing from responsibility. To state that the means of addressing this is for the IOC “to consider meaningful carbon compensation options” (17, p. 57) is also very weak, first because it only commits to considering these and second because carbon offsetting has been shown to be inherently problematic (55).

Where drivers and incentives are concerned, it is encouraging that the documents indicate the IOC has recognised the problems associated with overblown promises from OG host candidates and that alignment with existing plans, and recognition of cultural context, can result in achieving more by doing less. This should help reduce negative long-term unintended consequences arising from short-term intentional actions. However, while it is promising to see this approach, which should help to address the trend of bids containing ES promises that are not feasible, the 2021 Sustainability Report states that Paris and LA both made “substantial and unprecedented commitments” (50, p. 4) in sustainability, which risk unintended consequences and non-fulfilment.

It is also good to see cultural differences being acknowledged in the IOC's call for OG plans to align with a host's existing context and developmental direction in order to contribute to environmental (and other) issues. However, this is again not problem-free since it enables the IOC to sideline certain issues or intentions in favour of others so as to align with specific priority objectives, and the assertion that competing proposals cannot be directly compared allows for ambiguity in the awarding process and casts doubt over the purpose of the bid assessment process. There is also an argument that the reference to cultural context leaves the door open to awarding hosting rights to very environmentally damaging candidates where ES standards could be lowered on the basis of culture. Since the IOC has identified climate as “a matter of such critical importance that it requires special attention as a focus area in its own right” (48, p. 31), it could be reasonably expected that potential hosts will be considered only if they can demonstrate commitment to addressing climate change. Yet, there is significant speculation that Qatar or Saudi Arabia could be a future host, which suggests primacy of economic factors. This could be seen as relating to the TBL approach, whereby there is scope to prioritise a specific strand of sustainability over the others.

Caveats are again apparent in the terminology used, such as OM stakeholders being *encouraged* to embed sustainability in their operations, and the IOC referring to its “long-term strategic sustainability approach” (50, p. 8) rather than just to its strategy. Use of the word *approach* by the IOC here seems inappropriate since this is about governance by the IOC as leader of the OM. Since this is aligned with power relationships (degrees of control and influence) of the IOC as owner of the OG and leader of the OM, more robust leadership and strategic direction are needed rather than an *approach* that inherently allows for ambiguity.

Aspirations and intentions are clear throughout the documents. For example, in 2017 the IOC formed a Sustainability Working Group of European National Olympic Committees (NOCs), with the aim of subsequently establishing similar groups of other regional NOCs. However, it was noted in the 2021 Sustainability Report that attempts at extending this to other continents had been limited owing to the necessity of remote working in 2020. This disregards the fact that remote working can improve and facilitate communication and, as such, is not a credible reason for failing to extend the Sustainability Working Group initiative to other continents. This also provokes two further questions; (1) what about efforts to extend the initiative between 2017 and 2020, and (2) what progress has been made since? In answer to the first question, it is reasonable to assume that there was no progress or it would have been documented in the 2021 report. Unfortunately, with no Sustainability Report having been produced since 2021 (despite the stated commitment in the IOC Sustainability Strategy to biennial reports as well as shorter annual summaries), the second question remains unanswered. This in itself is revealing.

Progress has been similarly slow with the intention to embed sustainability in sourcing. Even where goods or services are sourced from official licensees or TOP partners, it is acknowledged that progress has not been as good as expected, which suggests again that power balances present a barrier. The suggested solution is to intensify training of buyers and to enhance engagement with partners, but this may not achieve the desired objectives if there is a conflict of interest between ES and the goals of TOP partners and licensees.

As previously noted, the IOC self-identifies as “the supreme authority of the Olympic Movement” (48, pp. 8, 22, 29) and, as such, the obligation to contribute to sustainability is acknowledged. Yet it would not be an unreasonable expectation for a worldwide supreme authority in any domain to lead rather than contribute to a global issue such as ES. The duty to lead is acknowledged in the 2021 Annual Report, which reports on separate speeches by Thomas Bach (IOC President) at the 2021 Sport Positive Summit and Prince Albert II of Monaco (Chair of the IOC Sustainability and Legacy Commission) at COP26 in which both stated: “As the leader of the Olympic Movement, the IOC has a responsibility to be part of the solution, and we have a responsibility to be ambitious about leading the change in the sporting world” (13, pp. 104, 105). It remains to be seen whether this is rhetoric, the repetition of which is intended to convince audiences and stakeholders of IOC commitment, yet it is promising that the emphasis is on leadership that reflects the reach and influence identified in Table 6 and the obligations identified in Table 7.

There have been tangible outputs from the IOC's collaborative work with ES stakeholders, such as the 2018 IUCN Sport and Biodiversity Guide. However, that this was produced as an IUCN publication with just a foreword from the IOC and an acknowledgement of partial funding, makes it difficult to ascertain the extent to which the IOC was actively involved.

In the 2021 Annual Report, it is recognised that the IOC “has flexibility to seize an opportunity in the best interests of the Olympic Movement, taking into account everything from socioeconomic and environmental factors to legacy, funding strategies and transport infrastructure” (13, p. 37). The inclusion of environmental factors is encouraging, but it is unclear how these factors would be prioritised and this means that again there is the likelihood of conflicts of interest.

There are many caveats presented in relation to opportunities to reduce the carbon footprint of corporate events such as the IOC Sessions (the general meetings of IOC members, held at least once annually). Since these events take place in various locations worldwide, it is claimed that travel and freight impacts are unavoidable, and the extent of impact depends on the locations of the OG and the IOC Sessions. Among other items, there is reference to furniture being freighted for IOC Sessions. Given that the IOC decides where the OG are to be held, perhaps the need for personnel travel and freight ought to be a consideration at the outset when evaluating the ES impact. However, perhaps more striking is the implicit assumption that IOC sessions must be held in different locations (with furniture freighted to these). Authentic commitment to ES would be demonstrable through having a consistent fully equipped venue in which to hold the annual IOC session and any extraordinary sessions. Better still, these could be held virtually as was the case for the 136th and 137th Sessions during the Covid-19 pandemic. This would also address the issue of inconsistent availability of sustainably sourced goods and services and variable reuse/recycling options in different locations.

Reliable data is problematic and there is evidence of manipulation of data, for example in the acknowledgment that since the IOC set a target for 50% reduction in CO2 emissions, reporting has not included such emissions from Olympic Broadcasting Services (OBS) Games time travel. Generation of reliable data is acknowledged as a key limitation. There are various reasons for this, including the cyclical character of IOC work, the length of time from candidature phase to post-Games reporting, erroneous perceptions of sustainability, and cultural and regulatory differences relating to ES in disparate countries. Simultaneously overseeing different OCOGs at different phases of planning, as well as cities interested in hosting, presents challenges in monitoring the implementation of IOC strategy and policies. However, these challenges must be overcome in order to develop clearer understanding of how impactful IOC activities actually are.

#### 4.1.5 The current state of play

The most recent Sustainability Report (50) is perhaps of greatest interest as this offers a snapshot of the current IOC intentions and objectives around ES. Table 8 shows the objectives from this report categorised according to the concepts and themes identified through the documentary analysis. Each objective is also identified by the sphere to which it belongs using the following notes in brackets at the end of the objective: Org – IOC as an organisation, OG – IOC as owner of the Olympic Games, OM – IOC as leader of the Olympic Movement.

**Table 8:** The latest IOC sustainability objectives coded by theme and associated first order concept.

Theme and associated first-order concept	Strategic objective
Networks and Knowledge Transfer	
Partnership and collaboration	Develop a sustainability strategy template appropriate for all NOCs to use, and in collaboration with Olympic Solidarity support NOCs in the implementation of sustainable practices (OM) Work with IFs whose sports are on the Olympic programme and NOCs for them to join the UN Sports for Climate Action Framework (OM) Work with partners, including UNEP, to develop a framework that will enable the Olympic Movement, athletes and fans to contribute to the development of the Olympic Forest (OM)
Expertise and experience	Assist and accelerate the transition to climate positive Olympic Games through the development of guidance and expertise for Interested Parties, Preferred Hosts and OCOGs, and the revision of relevant existing operational requirements (OG)
Sharing and applying best practice	Develop an expert network and regular forum to showcase best practice in sustainable innovation in sport infrastructure to inspire the Olympic Movement (OM) Work with and assist the Olympic Movement to leverage the information, best practices, guidelines and human capacity to implement sustainable actions through sport (OM)
Governance and Accountability	
Regulations, standards, codes, and compliance	Ensure that the IOC Sustainable Sourcing Guidelines are fully implemented across our supply chain while promoting respectful, sober, circular and regenerative models (Org) Work with IFs whose sports are on the Olympic programme and NOCs for them to apply, as a minimum, the basic level within the IOC Sustainable Sourcing in Sport guidelines to all procurement associated with the Olympic Games and Youth Olympic Games (OG/OM)
Targets, measurement, monitoring, and mitigation	Reduce our CO2 emissions in line with the Paris Agreement, with a 30 per cent reduction in our travel emissions by 2024 and additional measures targeting our digital activities, buildings and catering (Org) Work with IFs whose sports are on the Olympic programme to have a sustainability strategy in place by 2024 that sets goals, prioritises actions and tracks progress (OM) Support OCOGs and their partners in developing monitoring oversight of Olympic Games supply chains and construction workers’ rights as part of their human rights approach (OG)
Implementation: Analysis, evaluation, and review	Conduct a gap analysis across all the IOC's Olympic Games and Youth Olympic Games functional areas – and all Olympic Games phases – to identify areas where sustainability needs to be reinforced and formalised (OG)
Influence and Leadership	
Education, training, advocacy, and awareness-raising	Develop a comprehensive training programme, across all levels of responsibility, to increase staff competency in implementing the IOC Sustainability Strategy (Org) Work with OCOGs and partners to promote sustainable tourism and responsible consumption for Olympic Games participants, spectators and visitors to educate, create awareness and incite action on the ground (OG) Work with and support role models and influencers to raise awareness, educate and give visibility to sustainability through sport (OM)
Opportunities and Obstacles	
Expectations and obligations	Require that no permanent Olympic construction occurs in statutory nature and cultural protected areas and UNESCO World Heritage Sites and that the IOC, OCOGs and IFs work together to protect and enhance biodiversity within the host city/region and/or Games venues (OG)
Tangible and intangible outcomes and impacts	Create an Olympic Forest to support our climate positive objective while delivering long-term social and biodiversity benefits (Org)

The themes and concepts inherent in the objectives set out in Table 8 indicate that the IOC is becoming more focused on meaningful delivery and oversight of ES across the OM. Whilst this is encouraging, it is too soon to tell if the governance approach by the IOC will deliver on these objectives. An updated Sustainability Report should demonstrate the extent to which these have been achieved. Meanwhile, there are reasons to be cautious that arise from the earlier documents in the analysis sample and, specifically, in relation to the discourse used.

CPDA draws out existing ambiguities in the IOC agenda on ES, which need to be addressed for policy to be enacted that is appropriate, influential, and likely to yield positive ES outcomes. Furthermore, the level of ES commitment varied by theme, with discourse used in the theme of *Influence and Leadership* generally more committed as it described principles, the power of sport as a catalyst for raising awareness, and so on. The *Networks and Knowledge Transfer* theme demonstrates some evidence of internalisation of ES values by stakeholders as ES is incorporated into policy, strategy, and action throughout these networks. However, the discourse does not undisputedly offer clear evidence of the extent to which this may be attributed to IOC leadership. Discourse in the theme of *Governance and Accountability* also showed less commitment, with recommendations far more prevalent than requirements and many caveats to the IOC's level of control and influence that result in missed opportunities for the IOC to leverage its authority as owner of the OG and leader of the OM. Perhaps unsurprisingly it is in the theme of *Opportunities and Obstacles* that the discourse conveys the greatest evidence of caveats and provisos that infer a lack of commitment and accountability.

Results highlight the importance of policy discourse in shaping interpretations and implementation of ES initiatives that are meaningful and measurable and that seek to minimise negative unintended consequences. The ES impacts of the Games will be dependent upon the interpretation of policy and the culture of the host, which reflects the relationship between IOC policy guidelines (as captured in the Host City Contract Operational Requirements) and the implementation of these policy guidelines by diverse hosts. Ambiguous discourse, as is apparent in the vagueness of IOC policy statements, results in unintended consequences and undermines credibility. The approach demonstrated by the IOC will be reflected by other stakeholders (mimetic isomorphism), thus if language is ambiguous and policy involves recommendations rather than requirements, there will be less incentive for hosts and others to be authentic and adverse isomorphic processes will develop.

Power relationships have the potential to either facilitate or inhibit ES objectives, and there are inherent conflicts of interest that could result in greenwashing (16). The roles of a range of stakeholders are acknowledged in terms of power relationships, commitment, and accountability, and shifting balances of power between stakeholders may lead to conflicts of interest or offer opportunities for leverage. Accountability needs to be evident in all stakeholder relationships, with the IOC taking responsibility not only for its own ES actions but also holding to account the stakeholders it has authority over throughout the OM, including OG hosts, TOP partners, IFs, etc.

It is evident that the IOC has got far more involved with the ES agenda in recent years, but being visible in a space is not enough. There needs to be more ownership as the supreme authority of the OM, more accountability, for greater impact, otherwise it is likely that cynicism will remain about economic drivers taking precedence over environmental aims in the TBL of sustainability. It is clear that there are opportunities for the IOC to harness its leadership position more convincingly, through committed discourse and accountability, to better address ES and the climate emergency.

## 5 Discussion of key issues across the IOC's three spheres of activity

Atalay and Svagždienė (56, p. 67) claimed that “the sustainability strategy document of the International Olympic Committee is very important in terms of ensuring the balance between sports and the environment”, yet the discourse used in the strategy suggests that this is not being achieved.

There is a tendency for the IOC to downplay its power and influence when it comes to the OM with statements such as “in this context the IOC's level of direct control is limited” (48, p. 30), yet the IOC is referred to three times as “the supreme authority of the Olympic Movement” (48, pp. 8, 22, 29), and this is where impact would be greatest. Unfortunately, this undermines the achievements that have been made in the day-to-day operations of the IOC as an organisation, such as Olympic House, because it appears that rhetoric continues to outweigh meaningful action at a broader level. There are missed opportunities for the IOC to leverage its authority as owner of the OG and leader of the OM (6).

Of the three spheres of activity, there is evidence of stronger commitment to aspects of ES strategy in relation to the IOC as an organisation, for example progress on its delivery within the day-to-day operations in Lausanne is reviewed quarterly. However, reviews are conducted by an internal working group so it is possible that this process lacks objective governance that would demonstrate a higher level of genuine commitment.

In 2015 the IOC replaced its Sport and Environment Commission with a Sustainability and Legacy Commission and established a new department to oversee sustainability. This indicates a shift away from environmental concerns, which are arguably more challenging to address, towards the *Triple Bottom Line* of sustainability (economic, social and environmental factors) (23), allowing for obfuscation since any one or more of these factors can be invoked within strategy. A supplementary review of minutes from IOC Sessions predating the time frame of the documentary analysis presented here further attests to this shift, with the term environment being replaced by sustainability in frequency of usage during discussions in the years leading up to the Commission's name change.

The document sample showed that the new Commission is intended to not only fulfil an advisory capacity but also to provide a review function. This seems problematic in terms of objectivity since the terms of reference are likely to be either set or approved by the IOC, thus any reviews undertaken into ES or strategy implementation may be akin to the IOC marking its own homework.

Similar organisational changes relating to the situating of ES in IOC structures have occurred first with the amalgamation of sustainability with corporate development and brand, and more recently with sustainability (in its broader sense) being combined with “legacy, gender equality and inclusivity, and human rights into one department of Corporate and Sustainable Development” (46, p. 25). This breadth of important themes being combined in one department, in addition to the three strands of sustainability being merged, would make it difficult for all to be addressed especially as there will be competing priorities and potential conflicts of interest. Thus, the formation of this department is likely to result in unintended consequences that are detrimental to ES as a specific area of concern that is crucial in the fight against climate change. Furthermore, corporate development overlaps with sponsorship (as does financial sustainability) and this can be seen as antithetical to ES (53). It has been reported that future Games will involve more blatant product placement, which signals a shift towards further commercialisation of the IOC's own product and prioritisation of economic concerns.

The New Norm document is primarily focused on economic sustainability and viability as is evidenced by the breadth of the Legacy Strategic Approach as set out in the document. This results in missed opportunities to highlight ES benefits (e.g., in relation to right sizing of resources, which is listed under financial impacts). Various measures are outlined that are noted to “reduce both the construction and operational costs” (35, p. 26), yet there is no mention of this additionally reducing the environmental toll. This missed opportunity is repeated in relation to identification of measures to reduce the need for construction for the testing programme, Olympic village, and media centres.

This is further augmented by the lack of clarity over the specific sustainability objectives that are under consideration, which has arisen through the shift in terminology. For example, in relation to the requirement to minimise new construction, an overarching justification in terms of sustainability does not offer granular detail on both the environmental and economic elements of this. Cost savings may be more readily identifiable, but it would be helpful to know if there are environmental benefits to this approach and, if so, what these are. Olympic Broadcasting Services (OBS) cost benefit analysis identified notable cost savings through use of demountable reusable TV towers, but this was only specified in economic terms. This represents a missed opportunity to demonstrate positive environmental outcomes since the reuse of TV towers multiple times must yield carbon savings through reduced construction, manufacture of materials, etc.

Overall the document is focused overwhelmingly on cost savings and doesn't fully demonstrate where these also have positive environmental impacts, which arguably indicates a lack of true internalisation of ES by the IOC (36). Furthermore, this is likely to convey to stakeholders the relative importance of various aspects of sustainability, which in turn may lead to unintended consequences as economic aspects are prioritised over environmental concerns, especially as financial benefits such as cost savings as more tangible and thus are easier to demonstrate. The document as a cornerstone of the IOC's ES agenda could have emphasised the intangible benefits more, particularly in terms of ES and mitigating climate impacts that in turn affect health, participation, and so on.

Similarly, the 2018 Sustainability Report includes a great deal on governance and ethics but this content lacks specificity to ES and is focused instead on other aspects of viability, economic value, risk, and credibility. For example, the role of the Chief Ethics and Compliance Officer addresses risk and assurance, which seems to relate to the sustainability of the IOC itself. There is reference to the risk and assurance system and operational controls being aligned with the IOC's objectives, but it is not made clear whether this includes the 18 specific sustainability objectives identified in the Sustainability Strategy.

There is an extensive section on governance and ethics in the IOC Annual Report 2021, and this constitutes one over-arching theme from the documentary sample, hence it was important to refer to the IOC Code of Ethics to ascertain the extent to which ES is featured. The version accessed was the latest edition (January 2024) available from the IOC website (57). Overall, the guidance in the Code of Ethics relating to ES is weakly worded. In contrast to financial irregularities, which are explicitly forbidden and clear sanctions can be applied, there is no such clarity over what would constitute an environmental breach. ES expectations are not set out as rules (or articles) in the same way as other matters of ethical compliance. Rather, environmental considerations only appear in the section entitled *Basic Universal Principles of Good Governance within the Olympic Movement* – ipso facto these are not binding in the same way since principles are not the same as rules. This again highlights an inconsistency between the stated embeddedness of ES and the actual extent to which it is enshrined.

Whilst there is a great deal of positive rhetoric in the documents reviewed and, on the face of it, this points to commitment from the IOC to ES matters, there are also red flags that are identified through a CPDA approach. For example, the tendency for the IOC to downplay its power and influence when it comes to the OM (the realm in which impact arguably would be greatest) despite being the supreme authority in this sphere. An interesting paradox is also apparent in the tendency for the IOC to distance itself from accountability (e.g., where ES is stated to be the responsibility of OG hosts), which is contradicted by statements that appear to claim ownership of ES successes associated with the Olympic Games.

An apparent irony is that there is a clear first order concept of *opportunities* in the documents, yet there are several missed opportunities to highlight environmental positives arising from *New Norm* strategies such as minimising construction and reducing printed publications. The advantages of these guidelines are only presented as cost saving measures. There are also missed opportunities for the IOC to make more changes that would yield positive environmental outcomes, e.g., through minimising travel, having either a consistent venue for the IOC Sessions or holding these virtually.

In the 2018 Sustainability Report, it is claimed that changes relating to the OG are “testament to a willingness to re-examine established ways of working and find better ways of doing things” (17, p. 76). The missed opportunities identified above would suggest that this willingness is limited.

The reviewed documents contain evidence of key underpinning concepts, including knowledge transfer, power relations and policy discourse as identified above. Furthermore, data evidence the importance of stakeholders that form networks of interdependencies, and the capacity for unintended consequences to arise from policy. The power relationships between stakeholders have the potential to either facilitate or inhibit ES objectives, particularly when policy is open to interpretation. This highlights the need for clear and unambiguous IOC policy on ES that observes the following recommendations.

## 6 Policy implications and actionable recommendations

If climate change is to be addressed through sport, then leadership, commitment, accountability, and action are needed from the IOC and other stakeholders that have the power and influence to leverage wider social change for ES.

Policy must more explicitly identify mechanisms for enhancing knowledge transfer between hosts of major sports events and other stakeholders, taking into account cultural contexts and associated enablers and barriers.

Use of language in strategy and policy documents needs to be clear and non-contradictory to avoid obfuscation and ensure improved congruence.

Objective analysis of the IOC ES agenda is intended to identify opportunities for enhancing commitment to this cause and thereby contributing to improvements in ES outcomes of future mega-events.

A more fundamental rethink is needed to consider whether it is really necessary to hold such geographically widespread corporate events. The savings (in terms of both environmental impacts and economic factors) to be derived by holding these events in the same location or virtually would be significant. Given that the one objective kept open in the 2021 Sustainability Report was “include sustainability in corporate events”, surely there is a clear rationale for the approach to such events to be reconsidered. This would also facilitate achievement of the aim of consistency among corporate events.

Furthermore, in view of the considerable impact of OBS activities noted in Table 5, there is also a missed opportunity to more radically rethink how these operational delivery activities might be more effectively organised from an ES standpoint, e.g., with greater use of remote broadcasting. The relevance of this suggestion is strengthened by the acknowledgement that these activities are not explicitly captured in the Sustainability Strategy or CO2 emissions data yet are a material consideration.

## 7 Conclusion

Despite an emphasis on sustainability principles, values and intentions, and sport as an enabler of these, as well as proclaimed expectations of compliance, there is an implicit lack of accountability and commitment that is identified through the discourse used. Any ambiguity in policy discourse is open to subjective interpretations, which permits those enacting policy to shape it in line with their own interests and priorities. This highlights the importance of taking a CPDA approach to go beyond the surface of the policy discourse and identify deeper complexities and contradictions.

The inherent contradictions to be found within and across the document sample tend to undermine the positive aspects, cast doubt over authenticity and highlight a lack of policy congruence and coherence. These contradictions, since they do not exist independently of the IOC actors who produce the documents, may also point to an intentional manoeuvre by the IOC to not commit itself completely to ES and risk any consequent loss of autonomy or control. However, there are also signs of ES becoming more embedded in the IOC and the approach taken. The objectives identified in the 2021 Sustainability Report are clearer, more well-defined, are linked to the SDGs, and have criteria specified by which they will be measured. There is a caveat that the measurement criteria may be subject to change, which could indicate potential for moving the goalposts but seems more likely to be recognition that additional criteria may be needed.

The sphere with the most objectives (eight out of 15) is the IOC as leader of the OM, which arguably reflects the amount of work still to do in this sphere and the importance and potential impact.

The objectives largely reflect those that still required more work as noted in the Olympic Agenda 2020 Closing Report (18), emerging sustainability trends, and the challenges that have been identified. Since these objectives were for the period 2021–2024, it remains to be seen in the next report whether (and to what extent) they have been achieved.

### 7.1 Future research

To overcome challenges in monitoring the implementation of IOC strategy and policies, e.g., the cyclical character of IOC work and the length of time from candidature phase to post-Games reporting, it is important to look at this over a longer time frame. Thus, future work could build on the current study to review documents produced since the sample reviewed here. The current research could be further developed through an analysis of the ways in which IOC strategy and policy on ES has been implemented within the wider OM, e.g., by IFs, OCOGs or TOP partners. The policy discourse of future hosts and ES impacts arising from hosting, ES policies of TOP partners, IFs, and other stakeholders could also be explored and cross referenced against the IOC ES agenda.
